# Gorillas’ (*Gorilla g. gorilla*) knowledge of conspecifics’ affordances: intraspecific social tool use for food acquisition

**DOI:** 10.1007/s10329-020-00805-6

**Published:** 2020-03-12

**Authors:** Jacques Prieur, Simone Pika

**Affiliations:** 1 Department of Education and Psychology, Comparative Developmental Psychology, Freie Universität Berlin, Habelschwerdter Allee 45, 14195 Berlin, Germany; 2 Faculty of Human Sciences, Cognitive BioCognition, Institute of Cognitive Science, University of Osnabrück, Artilleriestrasse 34, 49076 Osnabrück, Germany

**Keywords:** Social tool use, Great apes, Knowledge of the social world, *Gorilla gorilla gorilla*, Cognition

## Abstract

The use of tools, long thought to be uniquely human, has now been observed in other animal taxa including several species of birds, non-primate mammals as well as some non-human primate species. Chimpanzees, one of humankind’s closest living relatives, exceed all other non-human animal species as they have been reported to use an exceptionally large toolkit. However, relatively little is known about the tool-use skills of the other great ape species. While the majority of tools described are inanimate objects, the use of social tools has received relatively little attention. Here we provide the first evidence of naturally occurring spontaneous exploitative behaviour of a conspecific as a social tool for food acquisition in non-human animals. We observed gorillas in captivity utilising a conspecific as a ladder to gain access to unreachable food. We discuss our findings in the light of other studies on social tool use and suggest the need for more nuanced interpretations of gorillas’ cognitive skills.

**Electronic supplementary material:**

The online version of this article (10.1007/s10329-020-00805-6) contains supplementary material, which is available to authorized users.

## Introduction

The complex use and manufacture of tools has shaped human evolution, culture, and social-cognitive abilities (e.g. Stout 2011; Toth and Schick 2015). Tool use has been defined as “the external employment of an unattached or manipulable attached environmental object to alter more efficiently the form, position, or condition of another object, another organism, or the user itself, when the user holds and directly manipulates the tool during or prior to use and is responsible for the proper and effective orientation of the tool” (Bentley-Condit and Smith 2010, p. 5). Although suggested for several decades as uniquely human (e.g. Oakley 1956), tool use has now also been reported in four phyla (Arthropoda, Chordata, Echinodermata, and Mollusca) and nine classes (crabs, insects, spiders, birds, fish, mammals, sea urchins, octopuses, and snails) (e.g. Bentley-Condit and Smith 2010; Shumaker et al. 2011). One of humankind’s closest living relatives, chimpanzees (*Pan troglodytes*), outrival all other non-human animal species, as they use an exceptionally large toolkit for diverse purposes (e.g. Boesch and Boesch 1990; Whiten et al. 2005; McGrew 2013). For instance, chimpanzees have been observed to crack nuts (e.g. Boesch and Boesch 1983), insert linear objects, mostly vegetation, into cavities to extract food items such as ants, honeybees or termites (Goodall 1964; Nishida 1973; Sanz and Morgan 2013), clip leaves in a variety of contexts (e.g. play, courtship, display; Nishida 1980; Kalan and Boesch 2018), and shake, drag or throw objects, usually sticks, in agonistic interactions with conspecifics (e.g. Goodall 1986; Call and Tomasello 2007). In contrast, relatively little is known concerning the tool-use skills of other great ape species, despite long-term observations of populations living in their natural environments (bonobos *Pan paniscus*: e.g. Kano 1982; Hohmann and Fruth 2003; Furuichi et al. 2015; gorillas *Gorilla* spp.: e.g. Schaller 1963; Tutin and Fernandez 1983; Breuer et al. 2005; orangutans *Pongo* spp.: e.g. van Schaik et al. 2003; Russon et al. 2009; Meulman and van Schaik 2013). Surprisingly, the majority of tool-use techniques reported so far involve inanimate objects (e.g. sticks, wooden hammers, stones), while relatively little is known about social tool-use abilities.

“Social tool” has been defined as a physical and/or psychological manipulation of an individual to achieve one’s own goal (e.g. Bard 1990; Völter et al. 2015). The degree of control between the social tool user and its social tools can vary according to four levels (Völter et al. 2015, 2016). Level 1 represents cases in which the social tool user fully controls its social tool in the same way as for an inanimate entity (e.g. pulling the arm of an individual to access the food that the latter is grabbing). In level 2, the social tool user has only partially physical control of the social tool: self-initiated and self-controlled actions of the social tool are required to achieve the goal (e.g. directing the arm of an individual toward a food item until the social tool has grabbed it, then pulling the arm back). Levels 3 and 4 represent cases of social tool use without any direct physical control. In level 3, the social tool user relies solely on the self-initiated and self-controlled actions of the social tool, who is treated as self-propelled machinery (e.g. giving a tool to an individual who will then act independently of the tool user to get the food item desired by the latter). In level 4, the social tool user solicits help from the social tool using communicative signalling (e.g. using a pointing gesture to direct the attention of an individual toward a food item to finally obtain it). To date, studies of non-human primates’ social manipulation abilities have mainly applied experimental paradigms and employed tasks that require identical and simultaneous or complementary and sequential actions typically of two interactants (e.g. see Völter et al. 2016 for review). These studies showed that several non-human primate species, including three great apes species (bonobos: Hare et al. 2007; chimpanzees: e.g. Crawford 1937; Schweinfurth et al. 2018; orangutans: Völter et al. 2015), tufted capuchin monkeys, (*Sapajus apella*) (Chalmeau et al. 1997; Mendres and de Waal 2000), and cotton-top tamarins (*Saguinus Oedipus*) (Cronin et al. 2005; Cronin and Snowdon 2008), socially manipulate conspecifics to achieve their own goals. For instance, Völter et al. (2015) found that orangutan mothers physically manipulated the bodies of their offspring to achieve their own goals (i.e. to obtain high-quality food). Depending on task demands, they exhibited relatively high degrees of flexibility, switching from exploitation to cooperation. In addition, two longitudinal studies focusing on tool use patterns showed social manipulation abilities in western lowland gorillas (*Gorilla gorilla gorilla*) (Gómez 1990, 1991) and Japanese macaques (*Macaca fuscata*) (Tokida et al. 1994). Gómez (1990, 1991) found that an infant hand-reared gorilla was able to recruit humans to help her obtain unreachable food rewards in a problem-solving task. In particular, the author reported that this infant developed a set of tactile gestures consisting in taking humans by their hand to appropriate locations or taking the hand of humans to external objects she wanted them to manipulate (e.g. latch of a closed door). In a provisioned troop of free-ranging Japanese macaques, three females who previously had learned to insert an inanimate entity (stick) to remove an apple from a horizontal pipe, extended previous experience in handling animate and reliable entities (her infants) to access the food (Tokida et al. 1994). The female macaques pulled their infants out of the pipe after they had caught the food, and one individual even actively pushed her infants into the pipe as though she were inserting a stick. However, so far relatively few reports have provided evidence of social tool use in naturally occurring spontaneous interactions. Studying social manipulations between conspecifics in real-life social contexts (i.e. close to contexts in which natural selection has acted) is very important, since it enables a better understanding of the selection pressures acting upon social manipulation strategies. “Agonistic buffering” is probably the most frequently reported social manipulation behaviour in non-human primates. It refers to male–male interactions in which one male handles a baby and/or a female to reduce the likelihood of aggression (e.g. Assamese macaques, *Macaca assamensis*: Kalbitz et al. 2017; Barbary macaques, *Macaca sylvana*: Deag & Crook 1956; gelada baboons, *Theropithecus gelada*: Dunbar 1984; olive baboons, *Papio anubis*: Strum 1984).

Here, we provide the first observations of naturally occurring spontaneous social tool use by non-human animals (gorillas) to obtain access to unreachable food.

## Methods

We observed a group of western lowland gorillas (*Gorilla gorilla gorilla*) living in the Apenheul Primate Park (Netherlands) from June to July 2017. During the observation period, the group was composed of eight females and five males between 3 and 42 years of age (mean = 13.84; SD = 13.36) (see Table 1). For a detailed description of the housing conditions see Prieur 2015.

**Table 1:** Individual characteristics of the study group of gorillas

Name	Age	Sex
Mature adult (over 20 years)		
Mintha	42	F
Mandji	41	F
Jambo	22	M
Young adult (12–20 years)		
Nemsi	15	F
Gyasi	14	F
Adolescent (7–11 years)		
Wimbe	8	M
Mapasa	8	M
Mfungaji	7	F
Juvenile (4–6 years)		
Mzungu	5	M
Chama	5	F
Tayari	5	F
Iriki	5	F
Infant (0–3 years)		
Jabari	3	M

Table 1 depicts the group composition of the study group as a function of name, age (in years), and sex. The age categories of subjects were based on Breuer et al.'s (2016) definitions for infants (0–3 years), juveniles (4–6 years), and adolescents (7–11 years), and on Stoinski et al.'s (2013) definitions for young (12–20 years) and mature (> 20 years) adults (F: female; M: male).

We collected daily behavioural data during four different 1.5 h sessions per day in the context of a study focusing on gorillas’ intraspecific communication signalling. Behaviours were videotaped using a full high-definition video camera (Canon Legria HF M506) equipped with an internal stereo microphone (sampling rule: focal animal sampling; recording rule: continuous recording; Martin and Bateson 1994). During these systematic observations, we were able to record three instances of an individual seemingly using a conspecific to obtain access to a branch of fresh oak leaves. We describe these three cases and a related tool-use observation in detail in the following paragraph.

## Results

Here, we report four observations of gorillas during the seasonal transition period from spring to summer. In Western Europe, this period is characterised by trees full of fresh leaves with flowers blooming. The gorilla enclosure of the Apenheul Primate Park includes several naturally occurring tree species such as beech (*Fagus sylvatica*), birch (*Betula* sp.), and oak (*Quercus* sp.). As they are attractive sources of food for the folivorous and opportunistically frugivorous western lowland gorillas (Tutin et al. 1991; Remis 1997; Rogers et al. 2004), the trees are protected by wire mesh to protect them against large folivore species. Access to fresh tree leaves, outside regular feeding events, is thus very difficult.

*Observation 1* We recorded the first behaviour of interest on 16 June at 01:37 p.m. Underneath an oak tree about 20 m high with a branch (2.5 m high) of fresh leaves, an infant male, Jabari, was sitting and feeding on a small wooden stick. An adolescent female, Mfungaji, approached Jabari from behind walking quadrupedally and looking at the oak tree branch. She briefly looked back as if to monitor her surroundings and then gently touched and grabbed (for definitions of gestures see Pika et al. 2003) Jabari with both hands. These tactile behaviours lacked effective mechanical force. Mfungaji looked at Jabari’s face when she touched and grabbed him, and with a relaxed face (for definitions of facial expressions see Van Hooff 1967; Gold 1991). Jabari also looked at her face and showed an open mouth threat while simultaneously grabbing her left wrist with his left hand. Mfungaji then put her right foot on Jabari’s right shoulder. Jabari opened his mouth more widely, thereby revealing his lower canines, and pushed her away using physical force (for differentiation of mechanically effective and ineffective behaviours see Pika 2008). As a result, Mfungaji turned away from Jabari without showing any resistance or opposition. Jabari closed his mouth, now expressing a relaxed face but still using a push gesture without mechanical force. Mfungaji moved even closer to Jabari (approximately 50 cm) while picking up the small wooden stick Jabari had previously used to forage. She then stood up in a bipedal posture, supported her own weight with the stick in both hands, and looked up into the out-of-reach oak tree. Jabari was still looking at her. After 3 s, Mfungaji sat down at a distance of 1 m from Jabari; both were still underneath the oak tree branch (see Electronic Supplemental Material ESM 1 for more details). An adult female, Gyasi, the focus subject of the next two observations, was sitting behind them with her back turned to Mfungaji, so that she could not have seen Mfungaji interacting with Jabari.

**Fig. 1:**
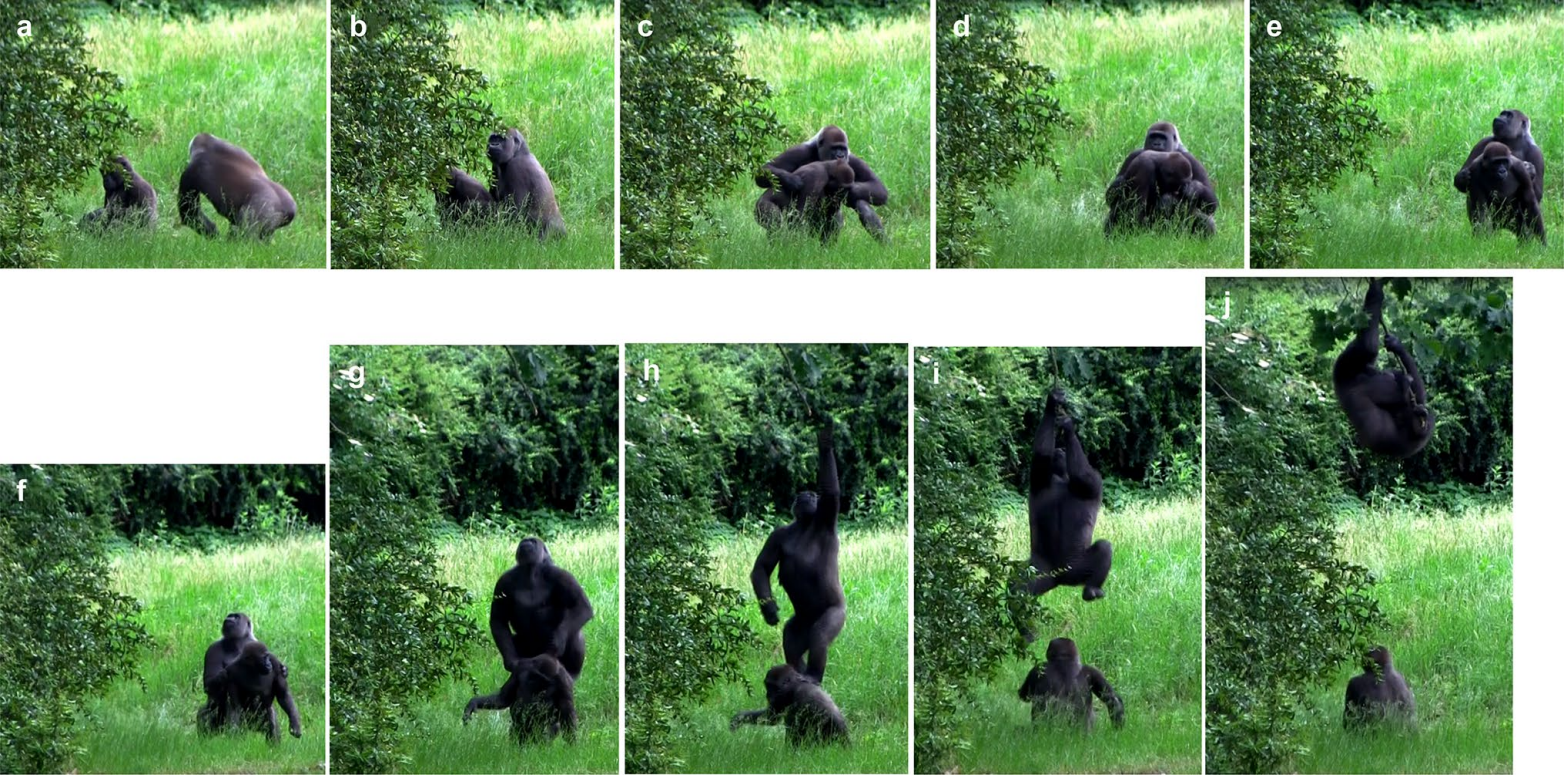
Behavioural sequence associated with Observation 2. An adult female Gyasi **a** approached a juvenile female Chama, **b** forcefully grabbed her under the shoulders with both hands, and **c** manipulated her to move her below the oak tree branch. Chama (**d**) shook her body and bit Gyasi several times. Gyasi (**e**) continued to firmly manipulate Chama while looking at the branch, **f** adjusted Chama’s position to place her just under the branch, **g** climbed onto her back, **h** stood bipedally on her and jumped toward the branch, and **i** grabbed it with both hands. Eventually, Gyasi (**j**) climbed up in the oak tree to get food access while Chama was looking at her

*Observation 2* We recorded the second behaviour of interest on 17 June 2017 at 10:39 a.m. A juvenile female, Chama, was sitting underneath the same oak tree and feeding on a small oak branch with fresh leaves, which had fallen from the tree. An adult female, Gyasi, approached Chama from behind (see Fig. 1a) and forcefully grabbed her under the shoulders with both hands (see Fig. 1b). She then pulled Chama with both hands (see Fig. 1c), walked beside her, and tried to move her under the oak tree branch (2.5 m high) full of fresh leaves. Gyasi showed a tight-lipped face and Chama an open mouth threat. Chama tried to escape by shaking her whole body and tried to bite Gyasi several times (see Fig. 1d). As Gyasi is more powerful than Chama, she forcefully grabbed Chama, thereby immobilising her, and pulled her closer to the branch. Simultaneously, she alternated her gaze between the branch and Chama (see Fig. 1e). In addition, Gyasi adjusted Chama’s position so that she was placed directly under the branch in a quadrupedal posture (see Fig. 1f). She grabbed Chama’s shoulders with both hands and then put her right foot and subsequently her left foot on Chama’s lower back to climb onto her back (see Fig. 1g). Gyasi now stood bipedally on Chama’s back, jumped toward the branch (see Fig. 1h), and grabbed it with both hands (see Fig. 1i). She then climbed up into the oak tree and started to feed on its leaves (see Fig. 1j; see also ESM 2 for more detail). After a foraging period of approximately 20 min, Gyasi jumped down from the tree without any leaves and hence did not share food with any of the other gorillas, including Chama.

*Observation 3* The third behaviour of interest was recorded on 17 June at 01:53 p.m. A mature adult female, Mandji, was lying on the ground underneath the same oak tree. Her daughter, the juvenile female Iriki, approached Mandji from behind and climbed onto her while looking at the oak tree branch, whereas Mandji remained static. Iriki then quickly jumped toward the branch, grabbed it with both hands, and climbed onto the oak tree to get access to fresh leaves while Mandji was looking at her. Iriki fed on leaves in the tree for about 20 s while collecting several branches with fresh leaves before jumping down the tree (see ESM 3 for more details). Iriki did not share any food with any of the other gorillas, including Mandji.

**Fig. 2:**
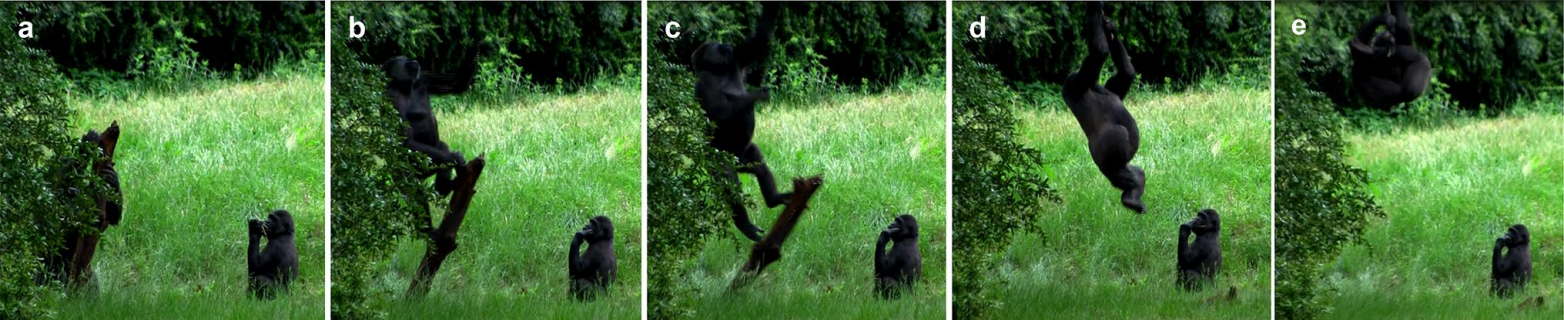
Behavioural sequence associated with Observation 4. The adult female named Gyasi (on the left) **a** erected a wooden log under the oak branch while firmly grabbing it with both hands, **b** climbed onto it while looking at the branch, **c** jumped toward the branch, **d** grabbed it with both hands, and **e** climbed onto the oak tree to get access to fresh leaves

*Observation 4* A related observation was recorded on the same day, 17 June 2017, at 02:40 p.m. The adult female, Gyasi, dragged a wooden log, which she had found approximately 100 m away, about 1.30 m and positioned herself underneath the same oak tree branch. Gyasi was in close proximity (ca. 1.5 m) to the infant male Jabari, who observed her during the whole subsequent sequence: Gyasi erected the wooden log while firmly grabbing it with both hands (see Fig. 2a), climbed rapidly onto it while looking at the oak tree branch (see Fig. 2b), jumped toward the branch (see Fig. 2c), grabbed it with both hands (see Fig. 2d), and (e) climbed onto the oak tree to get access to fresh leaves. Attracted by the noise caused by Gyasi, two adolescents, Mfungaji and Wimbe, and one juvenile, Tayari, approached the oak tree branch while observing Gyasi who was climbing up into the tree. Gyasi fed on leaves in the tree for approximately 20 min before jumping down the tree. Again, she did not bring down any leaves with her and did not share any food with any of her group members.

## Discussion

Here, we provide detailed descriptions of four behavioural events in which a gorilla explored different tactics to gain access to desired and out-of-reach food. Two different explanations may account for these observations.

First, the gorillas may have simply tried to get access to the food in the tree and took opportunistic advantage of conspecifics sitting underneath the oak trees to climb onto them. If this explanation is true, we predicted that gorillas would be frequently located close to the tree. In addition, we predicted that gorillas should show motivation (through gazes and actions) to act in order to achieve their foraging goal. Consistent with this explanation is the finding that all members of the gorilla group were observed to frequently position themselves underneath the oak tree and to look at the unreachable leaves. Furthermore, we observed gorillas using different behaviours to get access to the desired food: they (1) sat underneath the tree until some leaves fell opportunistically to the ground (e.g. on windy days), or (2) climbed onto an inanimate entity (a wooden log) or an animate entity (a conspecific) to increase their height and enable them to jump into the oak tree and grab a branch (2.5 m high). This explanation is supported by other studies showing that primates minimise search costs relative to resource gain (Chapman et al. 2012; Zuberbühler & Janmaat 2010). For instance, some species move to the closest available resource (tamarin monkeys *Saguinus mystax* and *S. fuscicollis*, Garber 1988; brown capuchin monkeys *Cebus apella*, Janson 1988) or plan efficiently their travel routes to optimise future resource gain (black capuchin monkeys *C. apella nigritus*, Janson & Byrne 2007; chacma baboons *Papio ursinus*, Noser & Byrne 2007; chimpanzees, Janmaat et al. 2014). Gorillas are opportunistic feeders and track food when seasonally available (mountain gorillas *Gorilla beringei beringei*, Watts 1998; Robbins & McNeilage 2003; western lowland gorillas, Rogers et al. 1990; Doran-Sheehy et al. 2009). Mountain and lowland gorillas present different feeding and ranging behaviours because they differ in their habitat ecology, especially in relation to the abundance and distribution of fruit resources (Tutin and Fernandez 1985; Williamson 1988; Remis 1994). Contrary to mountain gorillas, lowland gorillas live in habitats with more high-energy foods (fleshy and fibrous fruits) that vary spatially and seasonally and fewer low-quality foods (leaves, barks, and herbs) that are both available and consumed throughout the year. Thus, western lowland gorillas adjust their diet and activities to seasonal variation in fruit availability and decrease the time feeding while increasing the time spent travelling when food is abundant (possibly to locate dispersed fruit trees) (Tutin 1996; Masi et al. 2009).

Second, the gorillas took into consideration the conspecific’s affordance (i.e. conspecific’s physical and social characteristics such as his/her strength, size, rank, and/or kinship) to get access to the out-of-reach tree. If this explanation is true, we predicted that strength and/or rank would differ between the two animals involved: the stronger/higher-ranking animal climbing onto the weaker/lower-ranking one and not vice versa except when kinship was involved, which would favour cooperation and altruism toward genetic kin (e.g. Hamilton 1964; Lehmann and Keller 2006; West et al. 2007). In addition, we predicted that the supporting individual should be strong enough so that climbing onto and standing on his/her back/shoulders would be secure and tall enough to help reach the branch. These predictions are in line with our observations: the initiating individual was physically stronger and higher-ranking than the supporting animal (Observations 1 and 2) except when the individuals were closely related genetically (infant–mother dyad in Observation 3). Furthermore, the manipulated individual had the required strength and size to enable the initiating individual to increase her height and reach the branch (Observations 1–3). However, in Observation 4, the infant male underneath the oak tree branch was not strong or tall enough to help the actor reach the branch, whereas the wooden log was an adequate tool. In a similar vein, other studies report that other great ape species (bonobos: Grueneisen et al. 2017; chimpanzees: Call and Tomasello 2008; Fröhlich et al. 2016; orangutans: Völter et al. 2015), some monkey species (tufted capuchin monkeys: De Waal and Davis 2003; cotton-top tamarins: Cronin et al. 2005; Cronin and Snowdon 2008; long-tailed macaques, *Macaca fascicularis*: Overduin-de Vries et al. 2014), several non-primate mammals (goats, *Capra hircus*: Kaminski et al. 2006; hyaenas, *Crocuta crocuta*: Drea & Carter 2009), and some species of birds (western scrub-jays, *Aphelocoma californica*: Dally et al. 2006; keas, *Nestor notabilis*: Tebbich et al. 1996; Range et al. 2009) take into consideration the social affordances of conspecifics. For instance, chimpanzees are able to adjust their use of gesture in relation to distinct conspecific characteristics (age, sex, and kin relationship) (Fröhlich et al. 2016). Keas are able to manipulate conspecifics to gain access to enclosed food in relation to conspecifics’ rank status (Tebbich et al. 1996), and the social attention they pay to a conspecific exhibiting food-related behaviours depends on its age (Range et al. 2009). Furthermore, when facing experimental problem-solving tasks, chimpanzees can anticipate actions of dominant conspecifics by understanding the visual perception and knowledge of dominants (e.g. Call and Tomasello 2008; Hare et al. 2000, 2001). Chimpanzees can also choose to recruit the more effective of two conspecific partners based on their previous task success rate (Melis et al. 2006). By using a cooperative-solving task, Tebbich et al. (1996) reported keas’ intraspecific coercive instances of dyadic social tool use combining high differential (in strength and/or rank) with low social tolerance. The authors showed that, in dyadic test situations, three dominant keas aggressively approached their respective subordinate partners to force them to operate a lever opening a food apparatus without ever reciprocating. These experiments suggest that rank status would enable them to force “cooperation”.

We now discuss our observations focusing especially on current explanations emphasising the emergence and flourishing of diverse complex tool use behaviours in terms of ecological, social, and cognitive factors (e.g. van Schaik et al. 1999; Sanz et al. 2013; Koops et al. 2014). Our Observations 1, 2, and 3 showed individuals interacting with each other in a fundamentally different way depending on the level of control and motivation between the user and the social tool. Observation 3 is linked to the proposed level 1 of social tool use (Völter et al. 2015, 2016; Schweinfurth et al. 2018), with the initiating individual treating the social tool as a physical object (ladder) to reach the branch, while the social tool remained static, thus tolerating this act. Observation 2 is linked to the proposed level 2 of social tool use, with the initiating individual using mechanically effective and coercive behaviours toward the social tool, forcing the latter to perform self-initiated and self-controlled actions so that the initiating individual could climb onto and stand on the social tool’s back/shoulders to reach the branch. The initiating individual thus forced the social tool to cooperate, which is different from cooperation occurring upon communicative request that elicits a voluntary response from the social tool (e.g. Yamamoto et al. 2009, 2012). Observation 1 is linked to the proposed level 4 of social tool use, with the initiating individual using no coercive behaviour toward the social tool, and soliciting help from the latter using communicative signaling. However, in Observations 1 and 2, the social tools indicated their refusal to cooperate by performing different types of agonistic communicative behaviours such as an open mouth threat (e.g. van Hooff 1967; Bennett and Fried 1991; Dubois et al. 1991). This is in line with studies showing that gorillas do not seem to cooperate (Harcourt and Stewart 2007; Pelé et al. 2009). Our observations thus provide evidence of different levels of social tool use by gorillas in a foraging context. They also indicate that both interactants’ behaviours and physical and social characteristics (strength, size, rank, and/or kinship) may have important role to play in the process of social manipulation. These findings, along with the scarce literature on social tool use (chimpanzees, orangutans, and Japanese macaques: Völter et al. 2015; Schweinfurth et al. 2018; Tokida et al. 1994; keas: Tebbich et al. 1996), suggest that (1) the foraging context may be a useful candidate for investigating social tool use in the lesser-known and under-documented gorillas in terms of socio-cognitive skills, and (2) some features of social tool use might have evolved across different vertebrate lineages (primates and birds) through convergent evolution rather than only phylogenetic continuity. Our findings further emphasise the suggestion by Pika et al. (2019) that “reliable insights into the purpose cognitive abilities serve can only be gained by unravelling specific socio-ecological factors triggering their usage—a task demanding careful, knowledgeable observations of species living in their natural environments.” Deeper investigations of the ecological, social, and cognitive bases of social tool use in a wider range of primate species and in other lineages are necessary to improve our understanding of the evolutionary origins of human social manipulation.

## Conclusion

Here, we report the first observations of naturally occurring spontaneous social tool use in a non-human animal species (western lowland gorillas) to obtain access to unreachable food. Our findings reveal that gorillas are able to take into account conspecifics’ physical and social affordances (i.e. conspecific’s strength, size, rank, and/or kinship), shedding unprecedented light on gorillas’ knowledge of their social environment. Although based on only four observations, our results strongly emphasise the need for more nuanced interpretations of gorillas’ cognitive skills. In addition, they hopefully will inspire research into gorillas’ cognitive skills of individuals living in their natural environments.

Surprisingly, relatively little attention has been given to the comparative evolutionary approach studying social manipulation by human and other animals, despite the significance and permanent impact of manipulation and communication strategies (e.g. manipulation of public opinion through social media, marketing strategies, and consumption patterns) in the development of human societies. We need further research mapping out the phylogeny and ontogeny of social manipulation both within and between socio-ecologically relevant behavioural activities (e.g. foraging) to better understand the evolutionary mechanisms underlying the emergence and development of social manipulation.

## Electronic supplementary material

Below is the link to the electronic supplementary material.

Supplementary material 1 (WMV 7976 kb)

Supplementary material 2 (MTS 81815 kb)

Supplementary material 3 (WMV 2810 kb)

## Data Availability

The data sets supporting this article have been uploaded as part of the supplementary material.
